# Effect of fermented milk on upper respiratory tract infection in adults who lived in the haze area of Northern China: a randomized clinical trial

**DOI:** 10.1080/13880209.2021.1929344

**Published:** 2021-06-01

**Authors:** Hong Zhang, Junli Miao, Miya Su, Bryan Y. Liu, Zhenmin Liu

**Affiliations:** a Department of Respiratory Medicine, Beijing Chao-Yang Hospital, Capital Medical University, Beijing, China; b Dairy Research Institute, Bright Dairy & Food Co., Ltd, Shanghai, China; c College of Biotechnology, East China University of Science and Technology, Shanghai, China

**Keywords:** Probiotics, *Bifidobacterium animalis* subsp. lactis Bl-04, smoker

## Abstract

**Context:**

Upper respiratory tract infection (URTI) is the most common illness in humans. Fermented milk containing probiotics can mitigate URTI symptoms.

**Objective:**

This study tests the effect of fermented milk (Qingrun), a yogurt supplemented with *Bifidobacterium animalis* subsp. *lactis* Bl-04, on adults with URTIs who live in a haze-covered area in a randomized clinical trial.

**Materials and methods:**

A total of 136 subjects were enrolled in the study at the baseline and randomized to consume either control yogurt or Qingrun yogurt (250 g) once daily for 12 weeks. The duration and severity of URTI were evaluated by the Wisconsin Upper Respiratory Symptom Survey–24. Blood and faecal samples were collected at the baseline and post-intervention, to determine the changes of immune biomarkers.

**Results:**

Qingrun yogurt significantly reduced the incidence of the common cold (OR, 0.38; 95% CI, 0.17–0.81; *p* = 0.013) and influenza-like illness (OR, 0.32; 95% CI, 0.11–0.97; *p* = 0.045). Compared to the control yogurt, Qingrun yogurt significantly reduced the duration (1.23 ± 2.73 vs. 4.78 ± 5.09 d) and severity score (3.58 ± 7.12 vs. 11.37 ± 11.73) of URTI. In addition, the post-intervention levels of interferon-γ (139.49 ± 59.49 vs. 113.45 ± 65.12 pg/mL) and secretory immunoglobulin A (529.19 ± 91.70 vs. 388.88 ± 53.83 mg/dL) significantly increased in the Qingrun group, compared with those in the control group.

**Conclusions:**

Qingrun yogurt showed a protective effect against URTI in adults, suggesting that the use of yogurt with probiotics could be a promising dietary supplement for mitigating URTI.

## Introduction

Upper respiratory tract infection (URTI) is the most frequent illness in humans (Heikkinen and Ruuskanen 2006; Jespersen et al. 2015; Martineau et al. 2017). It can happen at any time but is most common in the fall and winter. Despite the benign nature of the disease, URTI causes a substantial economic burden on society in terms of medications, visits to physicians, and absenteeism (Heikkinen and Ruuskanen 2006). In addition to the common cold symptoms, such as a runny nose or plugged nose, sneezing, sore throat, itchy throat, cough, hoarseness, and head congestion, influenza-like illness (ILI) symptoms including headache, body aches, and fever are also commonly observed in URTI patients (Barrett et al. 2009). Most URTIs are caused by viruses and are self-limited, so antibiotics are rarely needed for their treatment unless bacterial complications occur (Heikkinen and Ruuskanen 2006).

Nutritional intervention that improves immune function can be a promising strategy to prevent or mitigate lung diseases including URTIs (Jespersen et al. 2015). Several studies have shown that yogurt and other fermented foods can improve intestinal and extra-intestinal health. The consumption of these foods might be useful in improving lactose intolerance, treating infectious diarrhoea, enhancing immune and anti-inflammatory responses, and reducing the incidence, duration, and severity of URTIs (Kok and Hutkins 2018). The protective effect of yogurt mainly comes from probiotics (Hao et al. 2015; Kok and Hutkins 2018), which have indicated various beneficial effects in protecting the hosts’ gastrointestinal and immune systems (Rijkers et al. 2010). According to several systematic reviews and meta-analyses of randomized controlled trials, probiotics can be used to prevent and treat URTIs in both children and adults (Wang et al. 2016; Möller et al. 2019).

It is well-established that the beneficial effects of probiotic supplements are both strain- and dose-specific (West et al. 2009; Rijkers et al. 2010). A previous study showed that *Bifidobacterium animalis* subsp. *lactis* Bl-04 with 2 × 10^9^ colony forming units (CFU) per day was useful in reducing the risk of URTI in healthy physically active adults (West et al. 2014). In this study, we investigated the efficacy of Qingrun, a yogurt supplemented with *Bifidobacterium animalis* subsp. *lactis* Bl-04, in mitigating URTIs in adults who live in a haze-covered area in northern China.

## Materials and methods

### Study design

**Figure 1.**
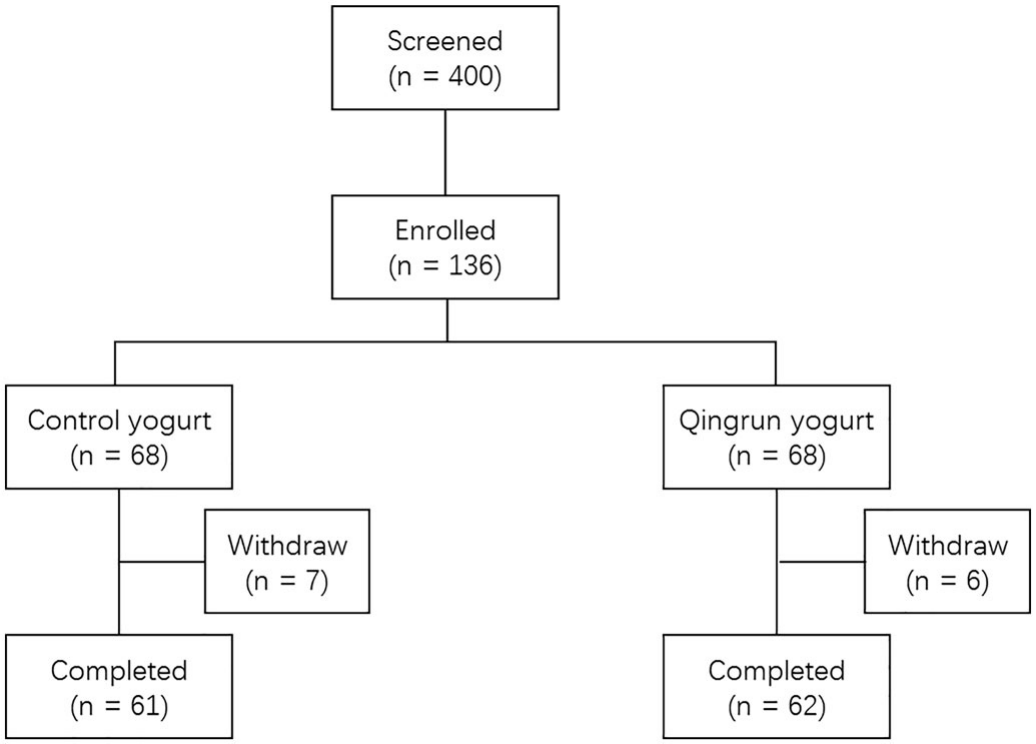
Flowchart of the clinical trial.

This randomized, double-blinded clinical trial was conducted in the Community Hospitals of Yayuncun, Zuojiazhuang, and Liulitun, in Beijing, China. In total, 136 subjects were initially enrolled in the study at the baseline and randomized to consume either the control yogurt or Qingrun yogurt (250 g) once daily for 12 weeks. The flowchart of the clinical trial is shown in Figure 1. The total dropout rate was 9.6%. Efficacy evaluations were performed at seven intervals: baseline, days 14, 28, 42, 56, 70, and 84. Informed consent was obtained from all subjects. This study was approved by the Institutional Review Board (IRB) of the Shanghai Nutrition Society and registered at chictr.org.cn (ChiCTR1900027437).

### Intervention

The test product was fermented milk packaged in a bottle. One bottle weighed 250 g. The yogurt was fermented from raw milk and favoured with an appropriate amount of white sugar, loquat jam, snow pear jam, whey protein powder, and food flavours. Lactic acid bacteria (*Bifidobacterium animalis* subsp. *lactis* Bl-04, *Lactobacillus casei*, *Lactobacillus bulgaricus*, and *Streptococcus thermophilus*) were also added in the Qingrun yogurt and the amount of *Bifidobacterium animalis* subsp. *lactis* Bl-04 was more than 2 × 10^9^ CFU/100 g. The control yogurt was processed similarly but without the addition of *Bifidobacterium animalis* subsp. *lactis* Bl-04, loquat jam, and snow pear jam. Both the Qingrun and control yogurts were manufactured in a BrightDairy Good Manufacturing Practice plant in Shanghai, China.

### Inclusion and exclusion criteria

According to the inclusion criteria, the study participants included male or female, 25–55 years old, white-collar workers (smoking or non-smoking) who lived in the haze area of northern China and had suffered from common cold 4–6 times in the past year. Subjects were excluded if they had a low immune system caused by chronic diseases, underwent treatment for gastrointestinal diseases, had diarrhoea, chronic rhinitis, or laryngitis, and had difficulties in breathing through the thoracic cavity or nose. These factors were similar to the URTI symptoms (e.g., chronic allergic rhinitis, asthma, chronic obstructive pulmonary disease). We also excluded those who were taking analgesic medicines such as aspirin or acetaminophen, had taken laxatives or other medication that could promote digestion 2 weeks before the study, had consumed milk or other foods that contained probiotics 10 d before the study, had a history of taking medicines that could prevent or inhibit URTI symptoms (e.g., antihistamine drugs, antitussive drugs, high-dose vitamin C), had taken medicines that could affect the immune response (e.g., antibiotics) 3 months before the study, had been vaccinated against URTI in the past 6 months or against other diseases in the past 15 d, had a history of alcohol or drug addiction, were pregnant or lactating, had participated in other clinical studies in the past 3 months, and were unwilling to comply with the study procedures.

### Evaluation of URTI

The duration and severity of the symptoms during a URTI episode were self-evaluated by the Wisconsin Upper Respiratory Symptom Survey-24 (WURSS-24). The WURSS-24 questionnaire consists of 1 overall severity item, 10 common cold-related symptom items, 3 ILI-related symptom items, 9 life quality items, and 1 overall change item (Barrett et al. 2009; Jespersen et al. 2015). Each item is rated on a 7-point Likert scale from very mild to severe. The definition of one URTI episode has been previously published (Jespersen et al. 2015). In brief, a common cold episode starts when ≥1 common cold symptoms are present for two consecutive days. An ILI episode starts if fever, in addition to one of the common cold symptoms, is present for two consecutive days.

### Immune biomarkers assessment

Blood and faecal samples were collected at the baseline and after the intervention. Interleukin 4 (IL-4), interleukin 8 (IL-8), interleukin 10 (IL-10), interferon-γ (IFN-γ), immunoglobulin A (IgA), immunoglobulin G (IgG), immunoglobulin M (IgM), and secretory IgA (sIgA) were determined using commercially available kits (Siemens Healthcare Diagnostics, Gwynedd, UK) according to the manufacturer’s instructions.

### Statistical analysis

The mean and standard deviation (SD) were reported for continuous variables; further, frequency and percentage were reported for categorical variables. The difference of incidence between the study groups was evaluated using a logistic regression model. A Chi-square test was used to examine the difference of categorical variables. Analysis of variance (ANOVA) and the Kruskal–Wallis test was used to determine the difference of continuous variables. In this study, the statistical analysis was performed using SAS 9.3 statistical software (SAS Institute Inc, Cary, NC, USA). A two-sided *p*-value <0.05 was considered statistically significant.

## Results

The baseline characteristics of the subjects who completed the study are summarized in Table 1. There was no significant difference in the characteristics between the two groups at the baseline. The number of URTI episodes in the past year ranged from 4 to six times. The percentage of smokers in the control and Qingrun groups were 32.8% and 35.5%, respectively.

**Table 1.** Baseline characteristics.

	Control yogurt *n* = 61	Qingrun yogurt *n* = 62	*p*-value
Male	35 (57.4%)	34 (54.8%)	0.777
Age (years)	35.7 ± 7.6	37.2 ± 9.2	0.310
Weight (kg)	68.8 ± 14.3	67.1 ± 12.2	0.479
Height (cm)	168.5 ± 8.3	166.8 ± 7.7	0.228
BMI (kg/m^2^)	24.0 ± 3.3	24.0 ± 3.0	0.946
Body temperature (°C)	36.3 ± 0.3	36.3 ± 0.2	0.458
Systolic blood pressure (mmHg)	112.9 ± 9.8	115.8 ± 8.2	0.081
Diastolic blood pressure (mmHg)	73.6 ± 6.3	75.5 ± 6.2	0.095
Number of URTI episodes in the past one year			
4 times	19 (31.2%)	14 (22.7%)	0.344
5 times	32 (52.5%)	32 (51.6%)	
6 times	10 (16.4%)	16 (25.8%)	
History of smoking	20 (32.8%)	22 (35.5%)	0.753
History of drinking	6 (9.8%)	6 (9.7%)	0.976

Data are presented as mean ± standard deviation or frequency (percentage).

Group deference was evaluated by using chi-squared test or analysis of variance (ANOVA).

URTI: upper respiratory tract infection.

During the 12-week intervention period, the incidence of common cold and ILI were 28 (45.9%) and 13 (21.3%) in the control group, and 15 (24.2%) and 5 (8.1%) in the Qingrun group, respectively (Table 2). Qingrun yogurt significantly reduced the incidence of common cold (odds ratio, 0.38; 95% confidence interval, 0.17–0.81; *p* = 0.013) and ILI (odds ratio, 0.32; 95% confidence interval, 0.11–0.97; *p* = 0.045). In addition, we observed a significantly higher incidence of common cold and ILI in the smokers compared to the non-smokers, with a *p*-value of 0.036 and 0.044, respectively (Supplementary Table 1).

**Table 2.** Incidence of upper respiratory tract infection throughout the study.

	Control yogurt *n* = 61	Qingrun yogurt *n* = 62	OR (95% CI)	*p*-value
Incidence of common cold	28 (45.9%)	15 (24.2%)	0.38 (0.17, 0.81)	0.013
Incidence of influenza-like illness	13 (21.3%)	5 (8.1%)	0.32 (0.11, 0.97)	0.045

Data are presented as frequency (percentage).

Group deference was evaluated by using logistic regression.

OR: odd ratios; CI: confidence interval.

We further explored the frequency of URTIs (including both common cold and ILI) during the study (Table 3). In total, 35 subjects (57.4%) reported one URTI episode in the control group, while 17 subjects (27.4%) reported one URTI episode in the Qingrun group. In the control group, six subjects (9.8%) reported two URTI episodes, while three subjects (4.8%) in the Qingrun group reported two URTI episodes. This difference was statistically significant (*p* = 0.0002). In addition, we observed a significant difference in the frequency of URTIs between smokers and non-smokers (*p* = 0.0001). In the haze days, the frequency of URTI in the smokers was higher than that in the non-smokers (83.3% vs. 64.5%), suggesting that smokers might be more likely to have URTIs in the haze days (Supplementary Table 2).

**Table 3.** Frequency distribution of upper respiratory tract infection (URTI) throughout the study.

Number of URTI episodes	Overall			Smoker			Non-smoker			Smoker *vs*. Non-smoker *p*-value
	Control yogurt *n* = 61	Qingrun yogurt *n* = 62	*p*-value	Control yogurt *n* = 20	Qingrun yogurt *n* = 22	*p*-value	Control yogurt *n* = 41	Qingrun yogurt *n* = 40	*p*-value	
None	20 (32.8%)	42 (67.7%)	0.0002	1 (5.0%)	11 (50.0%)	0.003	19 (46.3%)	31 (77.5%)	0.006	0.0001
Once	35 (57.4%)	17 (27.4%)		14 (70.0%)	9 (40.9%)		21 (51.2%)	8 (20.0%)		
Twice	6 (9.8%)	3 (4.8%)		5 (25.0%)	2 (9.1%)		1 (2.4%)	1 (2.5%)		

Data are presented as frequency (percentage).

Group deference was evaluated by using Kruskal–Wallis test.

Throughout the study, the duration and severity of the URTI symptoms were analyzed. Our results showed that the duration of URTI symptoms (days), severity score of URTI symptoms, and the duration of medication due to URTI (days) were significantly reduced in the Qingrun group compared with that in the control group (*p* < 0.0001, Table 4). No significant difference was observed in the duration of sick leave due to URTI (days) (*p* = 0.433). Additionally, we observed significant differences concerning the duration of URTI symptoms, severity score of URTI symptoms, and the duration of medication due to URTI between the smokers and non-smokers (*p* = 0.0009, 0.0003, and 0.0008, respectively).

**Table 4.** Duration and severity of upper respiratory tract infection (URTI) symptoms throughout the study.

	Overall			Smoker			Non-smoker			Smoker *vs*. non-smoker *p*-value
	Control yogurt *n* = 61	Qingrun yogurt *n* = 62	*p*-value	Control yogurt *n* = 20	Qingrun yogurt *n* = 22	*p-*value	Control yogurt *n* = 41	Qingrun yogurt *n* = 40	*p-*value	
Duration of URTI symptoms (days)	6.16 ± 5.46 7 (0–23)	1.95 ± 3.39 0 (0–13)	<0.0001	9.00 ± 5.21 9 (0–23)	3.27 ± 4.08 1 (0–13)	0.0005	4.78 ± 5.09 5 (0–16)	1.23 ± 2.73 0 (0–11)	0.0008	0.0009
Severity score of URTI symptoms	14.90 ± 12.25 18 (0–38)	5.79 ± 9.03 0 (0–27)	<0.0001	22.15 ± 10.10 20 (0–38)	9.82 ± 10.78 5 (0–27)	0.002	11.37 ± 11.73 9 (0–38)	3.58 ± 7.12 0 (0–24)	0.0009	0.0003
Duration of medication due to URTI (days)	3.64 ± 3.99 2 (0–14)	0.72 ± 1.79 0 (0–8)	<0.0001	5.70 ± 3.77 6 (0–14)	1.09 ± 2.18 0 (0–8)	<0.0001	2.63 ± 3.74 0 (0–13)	0.53 ± 1.54 0 (0–7)	0.003	0.0008
Duration of sick leave due to URTI (days)	0.52 ± 1.79 0 (0–10)	0.13 ± 0.59 0 (0–4)	0.433	0.90 ± 2.47 0 (0–10)	0.27 ± 0.94 0 (0–4)	0.502	0.34 ± 1.35 0 (0-–7)	0.05 ± 0.22 0 (0–1)	0.618	0.253

Data are presented as mean ± standard deviation and median (min-max).

Group deference was evaluated by using Kruskal–Wallis test.

After the intervention, the levels of IFN-γ and SIgA were significantly enhanced in the Qingrun group compared with those in the control group (*p* = 0.028 and *p* < 0.0001, respectively, Table 5). There were no significant differences in the levels of IL-4, IL-8, IL-10, IgA, IgG, and IgM between the two groups.

**Table 5.** Changes in the immune biomarkers.

	Biomarkers	Control yogurt *n* = 61	Qingrun yogurt *n* = 62	*p*-value
Baseline	Interleukin 4, IL-4 (ng/ml)	0.88 ± 0.29	0.87 ± 0.29	0.850
	Interleukin 8, IL-8 (pg/ml)	29.49 ± 16.05	29.97 ± 18.03	0.877
	Interleukin 10, IL-10 (pg/ml)	23.18 ± 1.43	23.06 ± 1.13	0.621
	Interferon γ, IFN-γ (pg/ml)	112.33 ± 59.95	114.49 ± 56.23	0.838
	Immunoglobulin A, IgA (g/L)	2.24 ± 0.53	2.35 ± 0.71	0.374
	Immunoglobulin G, IgG (g/L)	11.61 ± 2.26	11.65 ± 2.45	0.925
	Immunoglobulin M, IgM (g/L)	1.10 ± 0.42	1.13 ± 0.44	0.709
	Secretory IgA, SIgA (mg/dL)	384.27 ± 58.60	385.03 ± 23.93	0.952
Post-intervention	Interleukin 4, IL-4 (ng/ml)	0.85 ± 0.27	0.85 ± 0.27	0.947
	Interleukin 8, IL-8 (pg/ml)	27.78 ± 15.96	26.12 ± 15.66	0.561
	Interleukin 10, IL-10 (pg/ml)	22.77 ± 1.13	22.78 ± 0.96	0.935
	Interferon γ, IFN-γ (pg/ml)	113.45 ± 65.12	139.49 ± 59.49	0.028
	Immunoglobulin A, IgA (g/L)	2.26 ± 0.69	2.37 ± 0.74	0.401
	Immunoglobulin G, IgG (g/L)	11.66 ± 2.39	12.07 ± 2.29	0.338
	Immunoglobulin M, IgM (g/L)	1.11 ± 0.41	1.14 ± 0.48	0.780
	Secretory IgA, SIgA (mg/dL)	388.88 ± 53.83	529.19 ± 91.70	<0.0001

Data are presented as mean ± standard deviation.

Group deference was evaluated by using analysis of variance (ANOVA).

## Discussion

The main finding in this study was that fermented milk could reduce the incidence of common cold and ILI. In addition, the data evidenced shorter duration and less severity of symptoms during common cold and ILI episodes in the Qingrun group compared with the control group. This was consistent with the data from other clinical trials on probiotic interventions, which showed that probiotics were able to reduce the duration and severity of URTI symptoms (de Vrese et al. 2005; Guillemard et al. 2010; Smith et al. 2013; Fonollá et al. 2019). We also observed a shorter duration of medication, probably due to less severe URTI symptoms, though the duration of sick leave did not significantly change between the two groups. WURSS is an evaluative, illness-specific quality of life instrument, and the only validated questionnaire for self-reported symptoms of URTI (Barrett et al. 2009). In our study, we evaluated the duration and severity of symptoms during a URTI episode using the WURSS-24 questionnaire, which contains the addition of 3 ILI-related symptom items compared with the WURSS-21 questionnaire (Jespersen et al. 2015).

Previously, scientific research on probiotic supplements mainly focussed on one individual strain to simplify statistical analysis and data translation. Given the evidence that probiotic strains vary in a variety of neuroendocrine, immune and metabolic parameters, there is growing interest in the use of multi-strain probiotics (Goldenberg et al. 2015). Multi-strain probiotics are defined as supplements containing more than one bacterial strain, which are designed to reduce antagonism between strains, promote synergistic and additive effects, and customized for specific conditions (Timmerman et al. 2004; West et al. 2014). Multi-strain probiotics have clinical benefits for respiratory diseases (de Vrese et al. 2005; Ringel-Kulka et al. 2015). It was reported that yogurt containing the combination of *Streptococcus thermophilus*, *Lactobacillus bulgaricus*, and *Bifidobacterium animalis* subspecies *lactis* BB-12 improved the symptoms of URTI in children (Ringel-Kulka et al. 2015). In our study, we combined *Bifidobacterium animalis* subsp. *lactis* Bl-04, *Lactobacillus casei*, *Lactobacillus bulgaricus*, and *Streptococcus thermophilus* to develop a novel synbiotic yogurt. Our results highlighted the superior protective effect of this yogurt against URTI symptoms compared to the control yogurt without *Bifidobacterium animalis* subsp. *lactis* Bl-04. Indeed, *Bifidobacterium animalis* subsp. *lactis* Bl-04, alone, was found to be a useful nutritional supplement in reducing the risk of URTI in healthy physically active adults (West et al. 2014).

Loquat (*Eriobotrya japonica* (Thunb.) Lindl. [Rosaceae]) leaf and snow pear (*Pyrus nivalis* Jacq. [Rosaceae]) have been used to treat URTIs in traditional Chinese medicine for centuries. It was reported that flavonoids and triterpene acids isolated from loquat leaves inhibited oxidative stress and inflammation in mice with cigarette smoking-induced chronic obstructive pulmonary disease (COPD) (Jian et al. 2020a, 2020b). In addition, loquat leaf and snow pear attenuated airway inflammation and inhibited the allergic response in mice with ovalbumin-induced asthma (Lee et al. 2004; Kim et al. 2020). Daily consumption of snow pear juice improved plasma lipids and antioxidant capacity in smoking adults (Alvarez-Parrilla et al. 2010). It needs to be noted that the combination of several ingredients or nutrients may generate synergistic effects. Our study is the first to demonstrate the protective effects of a yogurt product containing these two botanical ingredients with probiotic bacterium against URTI, but whether a synergistic mechanism exists remains unknown.

It is well known that smoking is the main risk factor for premature mortality caused by cancer, cardiovascular disease, and COPD (Barnes et al. 2015). Smoking also seems to be a major risk factor for respiratory tract infection (Arcavi and Benowitz 2004). The mechanism of smokers’ increased susceptibility to infection is multifactorial, including changes in the host pulmonary structure and immune defense (Arcavi and Benowitz 2004). These structural changes include peribronchial inflammation and fibrosis, increased mucosal permeability, impaired mucociliary clearance, changed pathogen adhesion, and respiratory epithelial destruction (Dye and Adler 1994). It is reported that smoking affects both cellular and humoral immunity in humans and animals (Arcavi and Benowitz 2004). In our study, we observed significantly increased incidence, duration, and severity of common cold and ILI in smokers. However, it was still unknown whether the protective effect of the yogurt with probiotics against URTI was more prominent in smokers than in non-smokers.

Recent evidence suggests that air pollution is also a risk factor for cardiopulmonary disease (Brook et al. 2010; Guan et al. 2016). Fine particulate matter with an aerodynamic diameter of no more than 2.5 μm (PM2.5) is the main air pollutant. Long-term exposure to PM2.5 has detrimental effects on the respiratory system (Farina et al. 2011). In northern China, coal combustion in the winter always produces numerous pollutants including PM2.5, and the emission of pollutants has led to the emergence of haze in this area. In this study, we focussed on exploring the protective effect of the Qingrun yogurt in the subjects who lived in the haze-covered area of northern China. The data suggested that consumption of Qingrun yogurt in this area might help people to prevent or mitigate URTIs. Further, we observed that during haze days, the frequency of the occurrence of URTIs was higher in smokers than in non-smokers. This suggests that smokers were more likely to have URTIs in haze days. Our study was the first report that showed the influence of haze on the health status of smokers.

One of the study limitations is that we evaluated the incidence of ILI based on a self-reported WURSS questionnaire, which is clinically defined instead of being laboratory verified. Polymerase chain reaction-based nucleic acid detection is warranted in the future to verify the incidence and duration of URTIs. Another limitation is that the sample size of the sub-group analysis was relatively small. Due to this, only a trend was observed but no *p*-value could be calculated when comparing the frequency of URTIs in smokers versus non-smokers in haze versus non-haze days.

## Conclusions

We carried out a randomized clinical trial and showed the protective effect of fermented milk against URTIs in adults living in the haze-covered area of northern China. Qingrun yogurt was able to reduce the incidence, duration, and severity of URTIs, and improve immune biomarkers. Our results evidenced that yogurt with probiotics could be a promising dietary supplement to prevent and mitigate URTIs.
